# Feasibility of Transcatheter Closure of Large Secundum Atrial Septal Defect with Absent Superior or Inferior Rim

**DOI:** 10.1155/2022/2764296

**Published:** 2022-04-04

**Authors:** Hussein Abdulwahab, Mohammed Rassul Husain, Khalid A. Khalid

**Affiliations:** ^1^Department of Cardiology, Ibn Albitar Center for Cardiac Surgery, Baghdad, Iraq; ^2^Department of Pediatric Cardiology, Collage of Medicine, University of Basra, Basra, Iraq

## Abstract

**Introduction:**

Surgical closure of a large secundum atrial septal defect (ASD) with an absent superior or inferior rim is the standard method of management, but transcatheter closure of such a defect is possible and feasible.

**Objectives:**

To evaluate the feasibility, effectiveness, and safety of transcatheter closure of large secundum ASD with an absent superior or inferior rim through implantation of a cheatham platinum (CP) stent at the entrance of the superior vena cava (SVC) or inferior vena cava (IVC) into the right atrium (RA) to create a suitable rim for subsequent complete closure of the defect using a septal occluder. *Patients and Methods*. This case series was carried out at Ibn Al-Bitar Center for Cardiac Surgery, Baghdad, Iraq from 2014 to 2019, five patients underwent such transcatheter approach for closure of large secundum ASD with the absent superior or inferior rim by implantation of CP stent at the entrance of vena cave into the RA.

**Result:**

The ages and weights of patients who were enrolled in this study ranged from 9–31 years (15.2 ± 9 years) and 31.5–62 kg (42.6 ± 12 kg). Three patients had absent superior rims, and the other two had absent inferior rims. The *Q*_*p*_/*Q*_*s*_ was ranged from 1.9–3.2 (2.78 ± 0.29), and the mean pulmonary arterial pressure ranged from 22–29 mmHg (25.4 ± 3 mmHg). The defects with an absent superior rim were closed successfully by implantation of CP stents of 45, 45, and 39 mm to create a rim which supported the left atrial disc of 30, 38, and 32 mm atrial septal occluder (ASO), respectively, while large secundum ASD with an absent inferior rim could be effectively closed by implantation of two overlapping bare CP stents of 45 mm to create an IVC rim that supported 34 mm and 30 mm atrial septal occluder. *Conclusion and recommendation*. Transcatheter closure of large secundum ASD with absent superior or inferior rim is possible and effective by implantation of covered and bare CP stents at the entrance of SVC and IVC, respectively. Although these procedures are relatively difficult and challenging, especially in the closure of large defects associated with absent inferior rim, they carry a high risk of stent migration (8 zig, 45 mm), so we recommend using a CP-stent (10 zig, 60 mm).

## 1. Introduction

The secundum type atrial septal defect (ASD) is the fourth most common congenital heart defect with an incidence of 3.78 per 10,000 live births [1], corresponding to 5.9% of diagnosed congenital heart disease in children [2].

The ASD can now be treated by a transcatheter occlusion technique. Since the 1^st^ attempt in 1976 by King and Mills [3], transcatheter closure of secundum ASDs has evolved over the past 3 decades and has become an effective alternative therapy for most patients with ASD [4, 5].

Transcatheter closure of ASD has become the treatment of choice rather than surgery [6]. However, not all ASDs are anatomically suitable for transcatheter closure. ASD size should be limited and sufficient rims of interatrial septal tissue between the defect and adjacent structures are required to position the ASD device. Rims and the size of the ASD are crucial for the success of transcatheter ASD closure. The maximum diameter and dimension of the various rims of the ASD are essential for sizing and optimal placement of the device. Morphological variations of secundum type ASD are common and their recognition is crucial for the selection of suitability for percutaneous closure [7].

Transesophageal echocardiogram was done to assess size and rims in the different views (four chamber view ME 0°^,^ short axis view ME 45° and bicaval view ME 90–110°). AV rim and posterior superior rim in the four chamber view, aortic and inferoposterior rim in short axis view, and IVC rim in bicaval view were measured and documented. The views used for ASD assessment by TEE were as per the guidelines for echocardiographic assessment of atrial septal defect and patent foramen ovale of the American Society of Echocardiography and Society for Cardiac Angiography and Intervention [8]. A rim less than 5 mm was termed deficient or inadequate and absent if it was <1 mm.

The aim of this study was to evaluate our clinical experience in transcatheter closure of large secundum ASD with an absent superior or inferior rim by implantation of a CP stent in the entrance of vena cavae into the RA to create a septal fold by tight inflation of the stent.

## 2. Patients and Methods

A case series, which have been done at Ibn Al-Bitar Center for Cardiac Surgery, Baghdad, Iraq from 2014 to 2020 introduced a transcatheter approach for closure of large secundum ASD with an absent superior or inferior rim by implantation of covered and uncovered CP stents in the SVC or IVC, respectively, to create a suitable rim for subsequent complete closure by an atrial septal occluder.

Under local ethical committee approval and a fully signed patient's consent form, five patients (3 female and 2 males) with a mean age of 15.2 ± 9 years (ranged 9–31 years) and a mean weight of 42.6 ± 12 kg (ranged 31–62 kg) were included in this study.

Three patients were found to have large secundum ASD with an absent superior rim, and the other two had an absent inferior rim. They underwent an attempt of percutaneous ASD occlusion using an atrial septal occluder with CP-stent implantation.

All those patients refused surgery completely and insisted on transcatheter closure, so we were obligated to offer our new technique, explaining to them all the risks of such challenging procedures like stent migration, vascular injury, arrhythmia, and complete heart block. Meanwhile we called the surgical team to prepare all the requirements for an urgent open heart operation, as shown in Tables 1–3.

Assessment of the defect and its surrounding rims with the drainage of RUPV was based on transthoracic echocardiography (TTE) and transesophageal echocardiography (TEE) at the time of the procedure.

### 2.1. Device and Stent

The stent that was used in this study was the CP stent, 8 zig 39 and 45 mm, which is composed of heat-tempered 90% platinum and 10% iridium metal alloy, with metal wire arranged in a “zig” pattern that enables expansion up to 25 mm when inflated by a *Z*-med^TM^ balloon (Numed *R* Inc., NY, USA) size 18, 20, 23, and 25 mm × 40 mm.

The devices that were used for the closure of ASD in the study were the Amplatzer septal occluders sizes 30, 36, and 39 mm and the Occlutech septal occluder sizes 30 and 40 mm.

## 3. Results

Five patients (3 females and 2 males) were enrolled in this study. Their ages ranged 9 to 31 years (15.2 ± 9 years) and their weights were 31.5–62 kg (42.6 ± 12 kg). Three patients had absent superior rims, and two patients had absent inferior rims.

The *Q*_*p*_/*Q*_*s*_ ranged from 2.5 to 3.2 (2.78 ± 0.29), the mean pulmonary arterial pressure ranged from 22 to 29 mmHg (25.4 ± 3 mmHg).

**Figure 1:**
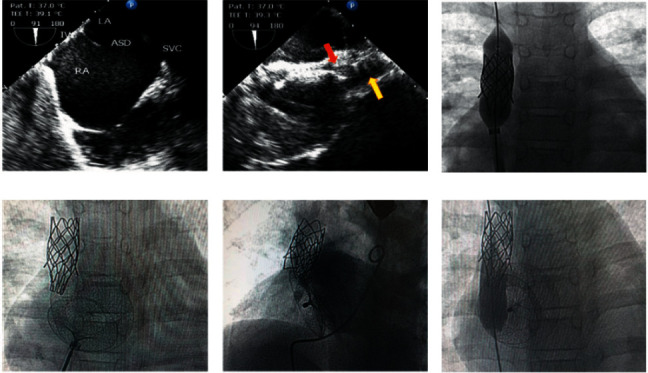
(a) Two-dimensional transesophageal echocardiography (TEE) imaging in long-axis bicaval view demonstrates a large secundum ASD with an absent rim near the SVC. (b) TEE in long axis view reveals the struts of the CP stent (yellow arrow) with the atrial septal occluder (red arrow) anchoring on the newly created superior rim by tight inflation of the covered-CP stent. (c) Cine imaging of the heart in the frontal projection demonstrates the inflation of the modified covered CP-stent 8 zig, 45 mm length by Z-med balloon size 22 × 40 mm. (d) Cine imaging of the heart in the frontal projection shows the deployed ASO in proper position with the projected part of the modified CP-stent out of RA disc before release. (e) Selective PA angiogram in four chamber view (LAO 35, cranial 35) demonstrates complete closure of the defect with no residual shunt. (f) Cine imaging of the heart in frontal projection demonstrates the inflated Z-med balloon (25 × 40 mm) slipped down in front of the septal occluder which did not capture the projected part of the modified CP stent, this balloon dilatation have been done three years later to achieve adult size of SVC.

The first patient was a 14 year-old-female with a large ASD secundum and an absent superior rim. She had dilated RA and RV, a *Q*_*P*_/*Q*_*S*_ of 2.5, and a mean pulmonary artery pressure of 23 mmHg. Successful closure of the defect was achieved by the implantation of a modified covered CP stent of 45 mm in the SVC. The modified CP stent was carried out by cutting half its circle into about 15 mm length to be projected into the RA and directed posteroinferiorly to be well aligned with the interatrial septum. The septal occluder which has been used to close the defect was an Amplatzer septal occluder size 30 mm with good deployment and total closure of the defect, but we found that the device was fixed on a fold which had been created by tight inflation of the stent and not anchored on the projected or sectioned part of the stent and that the device had never reached the stent (Figure 1). We started to use this new technique of creation of superior or inferior rim by tight inflation of a CP stent at the entrance of the superior or inferior vena cava without modification. We found this technique is easier than the first one which is even well aligned with the interatrial septum, but does not support the device.

**Figure 2:**
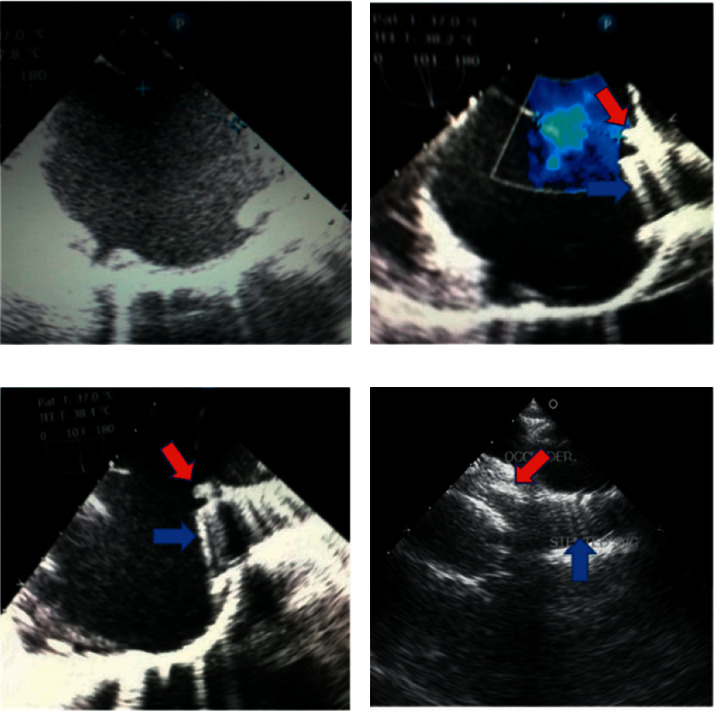
(a) TEE imaging in long-axis bicaval view demonstrates a large secundum ASD with an absent superior rim. (b, c) TEE imaging in long axis bicaval view demonstrates large secundum ASD with color follow mapping after inflation of the covered CP-stent creating a new superior rim (red arrow) with the clear struts of the stent (blue arrow). (d) TEE imaging in modified long axis bicaval view clearly demonstrates the struts of the covered CP-stent (blue arrow) creating a new superior rim (red arrow) which has been captured by two discs of the Occlutech septal occluder size 40 mm (red arrow).

The second patient was a thirty-one-year-old female with a very large ASD secundum associated with an absent superior rim and RV volume overload. The *Q*_*P*_/*Q*_*S*_ was equal to 2.8 and the mean pulmonary arterial pressure was 25 mmHg. She has been referred to us because of increasing dyspnea and exercise intolerance with palpitation. This patient underwent transcatheter closure of the defect which was carried out by the implantation of a covered CP-stent 8 zig, 45 mm at the entrance of the SVC into the RA to create new superior rim which would support the Occlutech septal occluder size 40 mm without residual shunt. Meticulous TEE assessment of the stent and the device revealed that the left atrial disc of the device was anchored on the septal tissue at the inferior edge of the stent without direct contact to the stent. Such a septal fold was confirmed after balloon inflation and before stent implantation, therefore we did not modify the stent in the second patient (Figure 2)

The third patient was 12 years old who had a large secundum ASD with an absent superior rim and RV volume overload. The *Q*_*P*_/*Q*_*S*_ was 2.9 and the mean pulmonary arterial pressure was 29 mmHg. The defect has been closed by the implantation of a 39 mm covered CP stent 8 zig in the SVC that resulted in the creation of the septal fold which supports the left atrial disc of a 36 mm Amplatzer septal occluder with no residual shunt. This patient develops an episode of SVT during the procedure that responds to I.V adenosine with no recurrence in the follow-up period.

**Figure 3:**
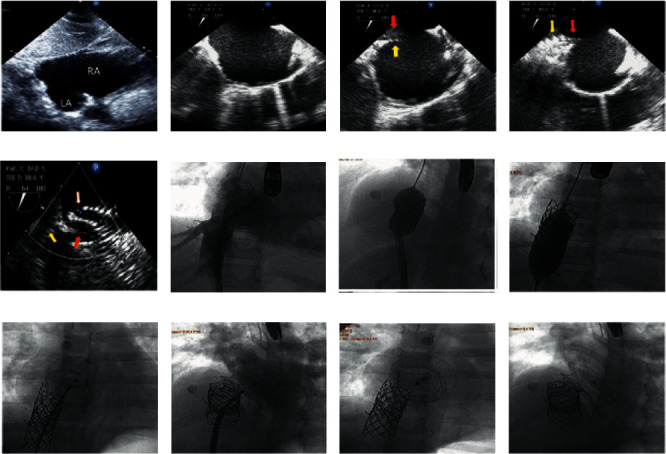
(a) Transthoracic echocardiogram, subcostal bicaval view demonstrates a large secundum ASD with an absent inferior rim. (b) TEE in long axis bicaval view shows large secundum ASD with absent inferior rim with right sided volume overload. (c, d) TEE in long axis view demonstrates the struts of the distal part of the inflated CP stent (yellow arrow) at the entrance of the IVC creating a new inferior rim (red arrow). (e) TEE imaging in a modified long axis view demonstrates struts of the inflated uncovered CP stent (yellow arrow) at the entrance of the IVC creating a new inferior rim (red arrow) which has been captured by two discs of the ASO (white arrow). (f) Cine angiogram of the IVC in frontal view demonstrates the uncovered CP stent mounted over the *Z*-med balloon size (25 × 40 mm) at the entrance of the IVC under the guidance of the TEE for proper position of the stent. (g) Cine imaging of the heart in four chamber view demonstrates the inflation of the uncovered CP stent, 8 zig, 45 mm, using *Z*-med balloon size (25 × 40 mm) at the entrance of the IVC into the RA under the guidance of the TEE. (h) Cine imaging of the heart in frontal view demonstrates the inflation of the second CP-stent 8 zig, 39 mm, overlapping with previous stent for proper fixation. (i) Cine imaging of the heart in the frontal projection demonstrates the proper deployment of the Amplatzer septal occluder size 34 mm before release. (j) RA angiogram in four chamber view through cook sheath 14F demonstrates the deployed septal occluder which was well aligned with IAS. (k) Cine imaging of the heart in aneteroposterior projection demonstrates both the two overlapping uncovered CP stents in the IVC with the proper position of the atrial septal occluder. (l) Cine imaging of the heart in four chamber projection demonstrates two overlapping uncovered CP-stents and the device which is well aligned with IAS under the guidance of the TEE, the device is not anchored the CP stent at all.

The forth patient was 9 years old male who had a large secundum ASD with an absent inferior rim (IVC) rim, dilated RA and RV, Q_*P*_/*Q*_*S*_ 2.5 and the mean pulmonary arterial pressure of 22 mmHg. The transcatheter closure of such defects is more difficult than those associated with an absent superior rim because the entrance of the IVC into the RA is almost always wider than that of the SVC, so the mounted uncovered CP stent 8 zig, 45 mm over the Z-med ^TM^ balloon size 25 × 40 mm migrated upward into the RA on the stiff Amplatzer guide wire after inflation, so the deflated balloon pushed upward inside the migrated CP stent and inflated to pull the stent downward to the IVC to be inflated tightly again, and another uncovered CP stent that was mounted over the *Z*-med ^TM^ balloon 25 × 40 mm pushed over the guide wire to be inflated half in the migrated stent and the remaining part inflated in the IVC in order to fix the 1^st^ stent (Figure 3). Meticulous TEE assessment of the stent with creating septal fold of the IVC rim and posteroinferior portion of the interatrial septum makes us proceed to compete closure of the defect using an Amplatzer septal occluder size of 34 mm with no residual shunt.

**Figure 4:**
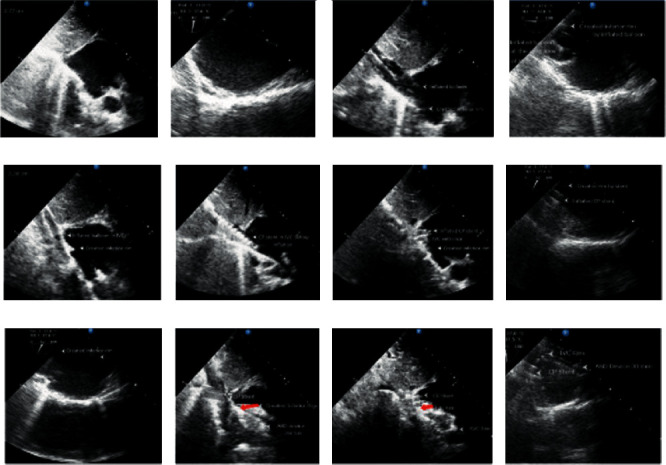
(a) Transthoracic echocardiorgraghy (TTE) imaging in subcostal view demonstrates a large secundum ASD with a totally absent inferior rim. (b) TEE imaging in long axis bicaval view demonstrates large secundum ASD. (c) TTE imaging in subcostal view demonstrates the inflated Z-med balloon with the created inferior rim (arrows). (d) TEE in modified oblique plane demonstrates the inflated balloon at the entrance of IVC with created inferior rim. (e) TTE imaging in the subcostal bicaval view demonstrates the inflated balloon with a created inferior rim. (f) TTE imaging in subcostal bicaval view shows the mounted uncovered CP stent at the entrance of the IVC before the inflation. (g) TTE imaging in subcostal bicaval view demonstrates the inflated uncovered CP stent at the entrance of the IVC with the created inferior rim. (h, i) TEE imaging in a modified long axis view (angle 67°) demonstrates the created inferior rim (upper arrow) by tight inflation of an uncovered CP stent at the entrance of the IVC (lower arrow). (j, k) TTE imaging in subcostal long axis view demonstrates the atrial septal occluder in proper position capturing the created inferior rim (red arrow). (l) TEE imaging in long axis bicaval view demonstrates the proper position of the atrial septal occluder capturing the new inferior rim (upper arrow) which has been created by inflation of uncovered CP stent (lower arrow) at the entrance of IVC.

**Figure 5:**
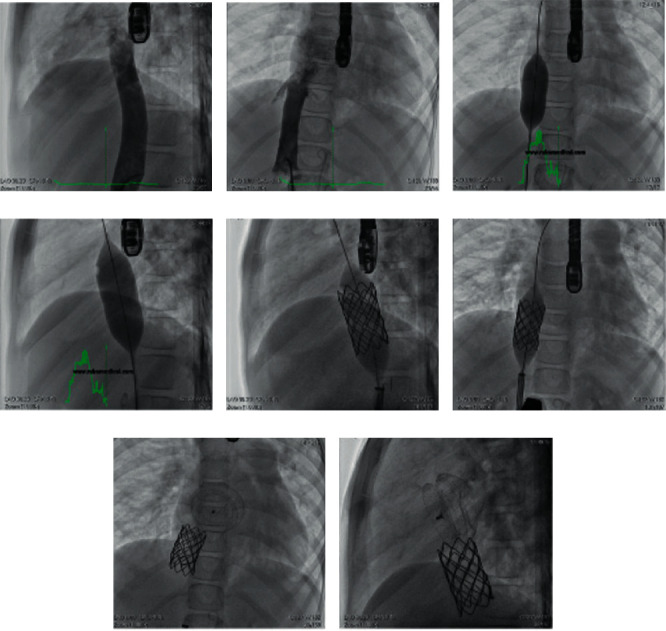
(a, b) IVC angiogram in frontal and lateral views demonstrates the diameter of the IVC at its entrance into the right atrium in order to select the proper size of the balloon. (c, d) Cine angiogram of the heart in frontal and lateral projections demonstrates the inflated Z-med balloon 25 × 40 mm at the entrance of the IVC to document that the inflated balloon can created an inferior rim. (e, f) Cine angiogram of the heart in frontal and lateral projection demonstrates the inflated uncovered CP stents at the entrance of the IVC into the right atrium. (g, h) Cine angiogram of the heart in frontal and lateral projection demonstrates proper implantation of uncovered CP stent at the entrance of the IVC, creating an inferior rim which makes the closure of the ASD secundum with an absent inferior rim possible and effective by the atrial septal occluder.

The fifth patient was a 10-year-old female with large secundum ASD with an absent inferior rim who had RV volume overload, *Q*_*P*_/*Q*_*S*_ of 3.2, and a mean pulmonary arterial pressure of 28 mmHg. The defect has been closed by the implantation of an uncovered CP stent 8 zig, 45 mm which was tightly inflated by a *Z*-med^TM^ balloon size of 25 × 40 mm at the entrance of the IVC, creating a septal fold at the site of the IVC rim and the posteroinferior part of the interatrial septum. TEE revealed the projection of the inferior rim over which the 30 mm Occlutech device anchored on this rim with total closure of the defect without residual shunt (Figures 4 and 5) (Supplementary Materials Videos 1–5).

### 3.1. Follow-Up

At the mean follow-up period (45 ± 13) months, four patients completed the follow-up period of 5 years, except the last patient had a 2-year follow-up. No one missed the follow-up.

The follow-up included clinical examination, electrocardiography, chest X-ray, and echocardiographic study, at 24 hours, 1 month, 3 months, 6 months, and annually after the procedure. TTE was performed at 24 hours, 1 month, and 3 months, while TEE was used at 6 months to evaluate the proper position of CP stent and ASO.

We kept all the patients on aspirin (5 mg/kg/day up to 100 mg/day) and clopidogrel (3 mg/kg/day up to 75 mg/day) for six months.

We encountered no significant complications acutely or on follow-up except one patient with absent inferior rim had stent migration at the time of implantation, including no patient developed complete heart block as we inflated the CP stent at the entrance of SVC into the RA.

## 4. Discussion

The ASO was designed to close ASDs with a deficient rim using two different mechanisms: stenting the defect with its connecting waist and eliminating any flow across the septum by the 2 flat discs. Because the right atrial disc is 8–10 mm and the left atrial disc is 12–16 mm larger than the connecting waist, a minimum of a 5 mm rim of atrial septum around the defect has been suggested as a prerequisite for the device closure with an ASO [9–11]. However, due to its design, it is believed that the ASO would not require an anterior rim for anchorage and the device would wrap around the posterior wall of the aorta. Furthermore, as long as the defect has equal or more than three sufficient rims, the device would seat well and effectively eliminate the shunt. A deficient anterior, inferior, or posterior rim as assessed by 2 dimensional echocardiography does not exclude the possibility that the defect could be closed using the ASO [8]. The findings of this study demonstrated that such defects associated with only one deficient rim and all defects had a distance of > 5 mm from vital cardiac structures(AV- valves, coronary sinus, right upper pulmonary vein) [8]. This is relatively comparable with our study in which either the superior or inferior rim is absent.

Our initial concept was to provide a stable rim to device anchorage in the case of transcatheter closure of a large secundum ASD with an absent superior or inferior rim. We have used a modified covered and bare CP stent in the SVC and IVC entrance into the RA by cutting part of the stent circumference (10–15 mm length) to be projected into the RA (Figures 1(d) and 1(f)) after inflation by the *Z*-med^TM^ balloon. We found that the device did not capture the projected part of the stent but it captured the newly created rim by tight inflation of the CP stent at the entrance of vena cavae into RA ((Figures 2(b)–2(d)), (Figure 3(e)), and (Figure 4(g), 4(h), and 4(l))), there after we change our technique in which the CP stent did not modified but just tightly inflated at the entrance of vena cavae into the RA. Before the stenting of the vena cavae we inflate *Z*-med^TM^ balloon to see whether the inflated balloon can create a new superior or inferior rim (Figure 4), thereafter we proceed with balloon mounted CP stent (covered stent at the entrance of SVC and bare stent at the entrance of IVC) to be inflated at the entrance of vena cavae (5–15 mm) into the RA creating a new superior or inferior rim, respectively ((Figure 2(b) and 2(c)), (Figures 3(c) and 3(e)), and (Figures 4(g) and 4(h))). All the five cases of large secundum ASD with absent rim (3 superior and 2 inferior) were successfully closed without taking oversized ASO just we selected devices 4–6.5 mm larger than the new diameter of the defect.

We thought that the mechanism of our technique could be explained by the concept of surgical repair of large secundum ASD with an absent superior or inferior rim where the initial stich in the floor of the IVC is tied. The large secundum ASD becomes almost slit-like before complete the repair, where a fold or flap will be formed at the site of the inferior rim which would be the future rim. Other stiches will be carried out at the anterior and posterior parts of this fold [12, 13], which also could be created by tight inflation of the bare CP stent at the entrance of IVC into the RA. Also, this concept could be applied to the absent superior rim which is easier than the absent inferior rim.

## 5. Conclusion

Although surgical repair of large secundum ASD with an absent superior or inferior rim is 1^st^ option.

But this case series study explain that such defects can be closed by transcatheter approach using tight inflation of the CP stent at the entrance of vena cavae into the RA to create new stable superior or inferior rim for anchorage of the device. Our result was comparable to that of surgical repair.

This technique is comparable to our initial technique for transcatheter closure for sinus venosus atrial septal defect with anomalous pulmonary venous drainage which was carried out in 2011 at Ibn Al-Bitar center for cardiac surgery, Baghdad, Iraq, and published in 2019 [14]. Therefore, we recommend using 3D printing and image fusion guidance in the transcatheter closure of large secundum ASD with an absent superior or inferior rim as done in catheter closure of sinus venosus ASD [15]. Although these procedures are relatively difficult and challenging, especially in the closure of a large defect associated with an absent inferior rim, they carry high risk of stent migration (8 zig, 45 mm), so we recommend using a CP- stent (10 zig, 60 mm).

## Figures and Tables

**Table 1:** Characteristics of the patients.

No.	Sex	Age/ys	Wt/kg
1	F	14	45
2	F	31	62
3	M	12	34.5
4	M	9	40
5	F	10	31.5

**Table 2:** Echocardiographic and hemodynamic characteristics of patients.

No.	Absent rim	ASD size in 4chamber view/mm	ASD size in bicaval view prestenting/mm	ASD size in bicaval view poststenting/mm	*Q*_*P*_/*Q*_*S*_	MPAP
1	Superior	24.3	30	24.3	2.5	23
2	Superior	33.2	35.8	30.6	2.8	25
3	Superior	24	27.6	23.3	2.9	29
4	Inferior	25.2	31.4	26.2	2.5	22
5	Inferior	25.5	28.2	22.6	3.2	28

**Table 3:** Characteristics of the procedure.

No.	Balloon size	Length of 8 zig CP stent	Device size	FT/min	PT/min
1	18/20	45	32	29.5	79
2	25/40	45	38	28.8	29
3	23/40	39	30	26	65
4	25/40	Two stents 45/45	34	42	113
5	25/40	39	30	51	118

## Data Availability

The data used to support the findings of this study are available from the corresponding author upon request.

## Supplementary Materials

Supplementary Materials Video 1: TEE in bicaval view demonstrates a large secundum ASD with an absent inferior rim. Video 2: TEE in bicaval view demonstrates an inflated balloon (yellow arrow) with the creation of an inferior rim (red arrow). Video 3: TTE in bicaval view demonstrates an inflated CP stent at the entrance of the IVC (yellow arrow) with a created inferior rim (red arrow). Video 4: TEE in bicaval view demonstrates the inflated CP stent (yellow arrow) and inferior rim (red arrow). Video 5: TTE in the bicaval view demonstrates the inflated CP stent (yellow arrow), with deployed occluder (blue arrow), capturing the inferior rim (red arrow).
